# Hodgkin-Reed-Sternberg Cells in Classical Hodgkin Lymphoma Show Alterations of Genes Encoding the NADPH Oxidase Complex and Impaired Reactive Oxygen Species Synthesis Capacity

**DOI:** 10.1371/journal.pone.0084928

**Published:** 2013-12-23

**Authors:** Maciej Giefing, Supandi Winoto-Morbach, Justyna Sosna, Claudia Döring, Wolfram Klapper, Ralf Küppers, Sebastian Böttcher, Dieter Adam, Reiner Siebert, Stefan Schütze

**Affiliations:** 1 Institute of Human Genetics, Christian-Albrechts University Kiel & University Hospital Schleswig-Holstein, Campus Kiel, Kiel, Germany; 2 Institute of Immunology, Christian-Albrechts University Kiel & University Hospital Schleswig-Holstein, Campus Kiel, Kiel, Germany; 3 Senckenberg Institute of Pathology, University of Frankfurt, Medical School, Frankfurt, Germany; 4 Department of Pathology, Hematopathology Section and Lymph Node Registry, Kiel, Germany; 5 Institute of Cell Biology (Cancer Research), University of Duisburg-Essen, Faculty of Medicine, Essen, Germany; 6 Second Department of Medicine, University Hospital Schleswig-Holstein, Campus Kiel, Kiel, Germany; 7 Institute of Human Genetics, Polish Academy of Sciences, Poznan, Poland; University of Barcelona, Spain

## Abstract

The membrane bound NADPH oxidase involved in the synthesis of reactive oxygen species (ROS) is a multi-protein enzyme encoded by *CYBA*, *CYBB*, *NCF1*, *NCF2* and *NCF4* genes. Growing evidence suggests a role of ROS in the modulation of signaling pathways of non-phagocytic cells, including differentiation and proliferation of B-cell progenitors. Transcriptional downregulation of the *CYBB* gene has been previously reported in cell lines of the B-cell derived classical Hodgkin lymphoma (cHL). Thus, we explored functional consequences of *CYBB* downregulation on the NADPH complex. Using flow cytometry to detect and quantify superoxide anion synthesis in cHL cell lines we identified recurrent loss of superoxide anion production in all stimulated cHL cell lines in contrast to stimulated non-Hodgkin lymphoma cell lines. As *CYBB* loss proved to exert a deleterious effect on the NADPH oxidase complex in cHL cell lines, we analyzed the *CYBB* locus in Hodgkin and Reed-Sternberg (HRS) cells of primary cHL biopsies by *in situ* hybridisation and identified recurrent deletions of the gene in 8/18 cases. Immunohistochemical analysis to 14 of these cases revealed a complete lack of detectable CYBB protein expression in all HRS cells in all cases studied. Moreover, by microarray profiling of cHL cell lines we identified additional alterations of NADPH oxidase genes including *CYBA* copy number loss in 3/7 cell lines and a significant downregulation of the *NCF1* transcription (p=0.006) compared to normal B-cell subsets. Besides, NCF1 protein was significantly downregulated (p<0.005) in cHL compared to other lymphoma cell lines. Together this findings show recurrent alterations of the NADPH oxidase encoding genes that result in functional inactivation of the enzyme and reduced production of superoxide anion in cHL.

## Introduction

The NADPH oxidase is a multi-protein enzyme consisting of two membrane bound subunits, the p22-phox and gp91-phox and three cytoplasmic subunits, the p47-phox, p67-phox and p40-phox [1]. These proteins are encoded by the *CYBA* (16q24.3), *CYBB* (Xp11.4), *NCF1* (7q11.23), *NCF2* (1q25.3) and *NCF4* (22q12.3) genes, respectively. The function of NADPH oxidase has been historically associated predominantly with phagocytes and their role in host defense. Phagocytic cells undergo a process called oxidative burst to generate large amounts of superoxide anion and other secondary ROS (reactive oxygen species) of microbicidal function. In line with this observation, genetic defects in any of the NADPH oxidase genes cause impaired functionality of phagocytes, immunodeficiency and manifest in chronic granulomatous disease characterized by recurrent and severe infections including pneumonia, infectious dermatitis or osteomyelitis (Online Mendelian Inheritance in Man database - OMIM): *CYBA* 233690, *CYBB* 306400, *NCF1* 233700, *NCF2* 233710, *NCF4* 613960) [2,3].

Beside the role in host defense, the NADPH oxidase is used by non-phagocytic cells to synthesize small amounts of ROS [4-6], that rather than having microbicidal properties modulate signaling pathways involved in differentiation, cell cycle regulation and apoptosis. In hematopoietic cells of *Drosophila*, for example, scavenging ROS was demonstrated to delay differentiation of progenitors into mature blood cells [7]. In humans, reduced NCF4 protein expression impaired normal B-cell functionality by hampering MHC class II antigen presentation [8]. Moreover, the link to B-cell lymphoma pathogenesis is suggested by genotyping studies where functional polymorphisms of the *CYBB* gene were shown to influence outcome in non-Hodgkin lymphoma patients [9-11]. The regulatory role of NADPH oxidase derived superoxide was demonstrated also in murine B-cells where mice knockouts for the CYBB protein homolog showed downregulation of the cell cycle arrest inducing p27^Kip1^ protein and higher B-cell proliferation [1].

In light of the above and intrigued by the transcriptional downregulation of the *CYBB* gene in classical Hodgkin lymphoma (cHL) cell lines reported in our previous study [12], we investigated here the functionality of the NADPH oxidase complex in cHL cell lines. We show impairment of the NADPH oxidase function and identify alterations within genes encoding components of the NADPH oxidase complex as potential molecular mechanisms resulting in the inactivation of the enzyme.

## Results

### Copy number analysis of the CYBA, CYBB, NCF1, NCF2 and NCF4 genes and mutation screen of the CYBB gene shows frequent deletion of CYBB in cHL

Our recent observation of *CYBB* downregulation in cHL cell lines led us to analyze these cell lines for deletions of genes encoding components of the NADPH oxidase complex. By mining SNP microarray data we identified deletions of *CYBB*, that is located on the X chromosome, in 2/7 (29%) cHL cell lines including a heterozygous deletion in the L540 cell line derived from a female cHL patient and the previously described homozygous deletion in KMH2 [12]. To identify the putative second hit in *CYBB* in the heterozygous L540 cell line and further mutations in the other five cell lines (excluding KMH2) - out of which four are derived from male patients - we sequenced the entire coding sequence and exon-intron boundaries of the gene, but no mutations were identified. We extended the analysis to a copy number screen of the *CYBB* gene in 18 primary cHL cases and analyzed lymph node cryosections by combined immunophenotyping and interphase cytogenetics. Altogether we identified 8/18 (44%) cases with a signal constellation indicative for deletions of the *CYBB* gene with regard to the sex of the patients and the ploidy of the cases. These included six deletions restricted to the p arm of the X chromosome harbouring the *CYBB* locus with retained X centromere, and two deletions of the entire X chromosome. No cases with complete *CYBB* loss were identified.

Moreover, using the SNP microarray data we identified alterations of the *CYBA* locus in 3/7 (43%) cHL cell lines including losses in HDLM2 and L540 and loss of heterozygosity (LOH) in the KMH2 cell line. LOH of the *NCF2* locus was observed with a similar frequency, that is 3/7 (43%) cell lines, in L428, KMH2, UHO1, and of the *NCF4* locus in one cell line, namely UHO1 (Table 1). No copy number losses were identified for the *NCF1* gene. 

**Table 1:** Alterations of the NADPH oxidase complex genes in cHL cell lines based on SNP 6.0 microarray profiles.

	CYBA	CYBB	NCF1	NCF2	NCF4
L428			Hypermethylated [16]	LOH	
HDLM2	loss		Hypermethylated [16]		
KMH2	LOH	bi-allelic loss	Hypermethylated [16]	LOH	
L1236			Hypermethylated [16]		
SUPHD1			na		
UHO1			Hypermethylated [16]	LOH	LOH
L540	loss	loss	na		

LOH - loss of heterozygosity; na – not analyzed.

Taken together, beside frequent losses of *CYBB*, other NADPH oxidase encoding genes are recurrently targeted by genetic alterations in cHL. 

### mRNA expression of NADPH oxidase subunits is significantly downregulated in cHL cell lines and primary biopsies

**Figure 1:**
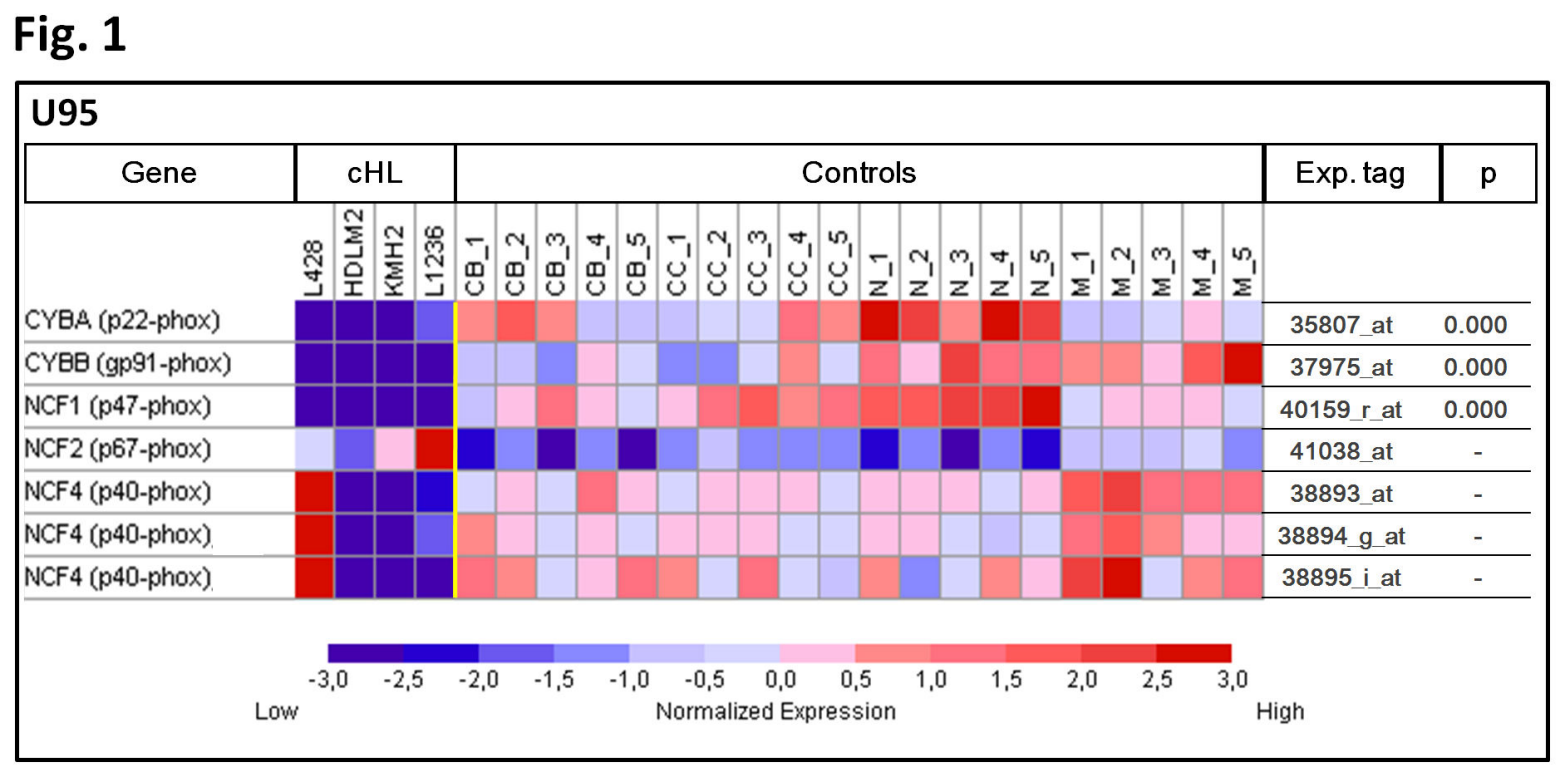
Microarray expression analysis of NADPH oxidase encoding genes CYBA, CYBB, NCF1, NCF2 and NCF4. Relative expression of the five genes in 4 cHL cell lines and 20 normal B-cell samples. CB - centroblasts, CC - centrocytes, N- naive B-cells, M - memory B-cells. The p value is given only for genes showing significant changes in expression between cHL and controls. Based on published Genechip data [13].

To analyze if the genomic losses of the NADPH oxidase encoding genes correspond to decreased mRNA expression of these genes we used published gene expression data sets of four cHL cell lines and 20 normal B-cell samples, representing centroblasts, centrocytes, naive B-cells and memory B-cells [13]. As shown in Figure 1, besides the downregulation of *CYBB* reported before, we also observed significantly lower expression of *CYBA* (p<0.001) and complete downregulation (absent calls) for *NCF1* (p<0.001) in the four cHL cell lines as compared to the B-cell controls. For the *NCF4* gene lower expression was observed in 3/4 cHL cell lines (Figure 1). This confirms that the NADPH oxidase genes are deregulated at mRNA level in cHL cell lines and suggests that other mechanisms than deletions must be responsible for the observed loss of *NCF1* expression.

**Figure 2:**
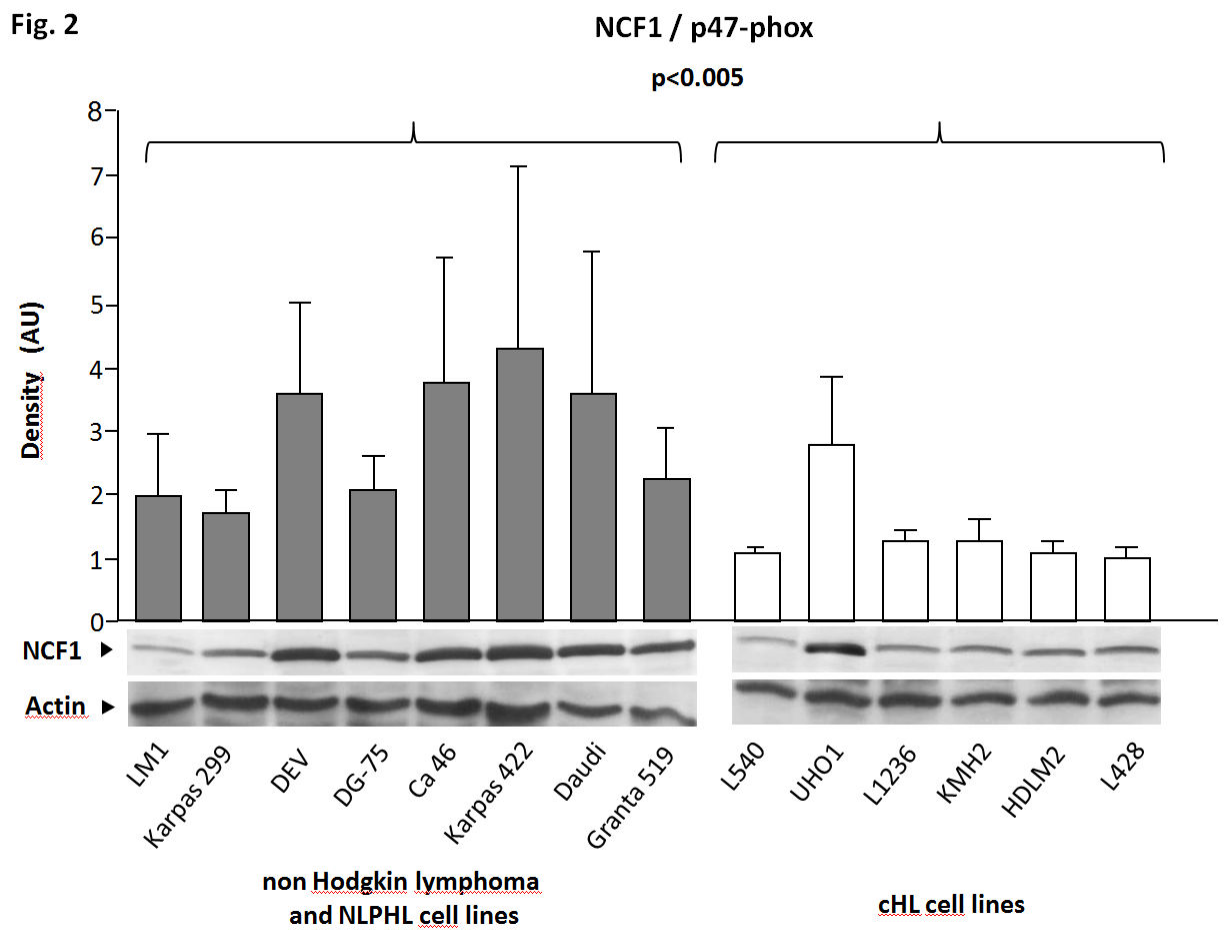
Western blot analysis of the NCF1 protein. NCF1 protein expression in 8 non-cHL lymphoma cell lines (LM1, Karpas 299 (CD30^+^), DEV (CD30^+^), DG-75, Ca 46, Karpas 422, Daudi, Granta 519) and 6 cHL cell lines (L540, UHO1, L1236, KMH2, HDLM2, L428 - all CD30^+^). AU – arbitrary units after normalization to actin signal strength. Each bar presents the mean result of 6 independent Western blots and is exemplified by a blot presented below. cHL cell lines show significantly lower (p<0.005) expression of the NCF1 protein as compared to the control cell lines.

In order to investigate whether loss or downregulation of the NADPH complex is also a feature of uncultured primary HRS cells we extended the analysis to microdissected HRS cells from 12 primary cHL cases [14]. As compared to 25 normal B-cell samples, significantly lower expression of the *NCF1* gene was observed in HRS cells (p=0.006 probe set 223724_s_at and p<0.001 tag 214084_x_at) but not of the *CYBA*, *CYBB*, *NCF2* and *NCF4* genes. In line with this finding, we observed significantly lower expression of NCF1 on protein level in cHL cell lines compared to non-Hodgkin lymphoma cell lines (p<0.005) (Figure 2). 

Thus, combining genomic data and expression analysis on mRNA as well as protein level provides a strong rationale for the hypothesis of NADPH oxidase impairment resulting in reduced ROS synthesis capacity in cHL.

### CYBB protein is absent in HRS cells of primary cHL biopsies

**Figure 3:**
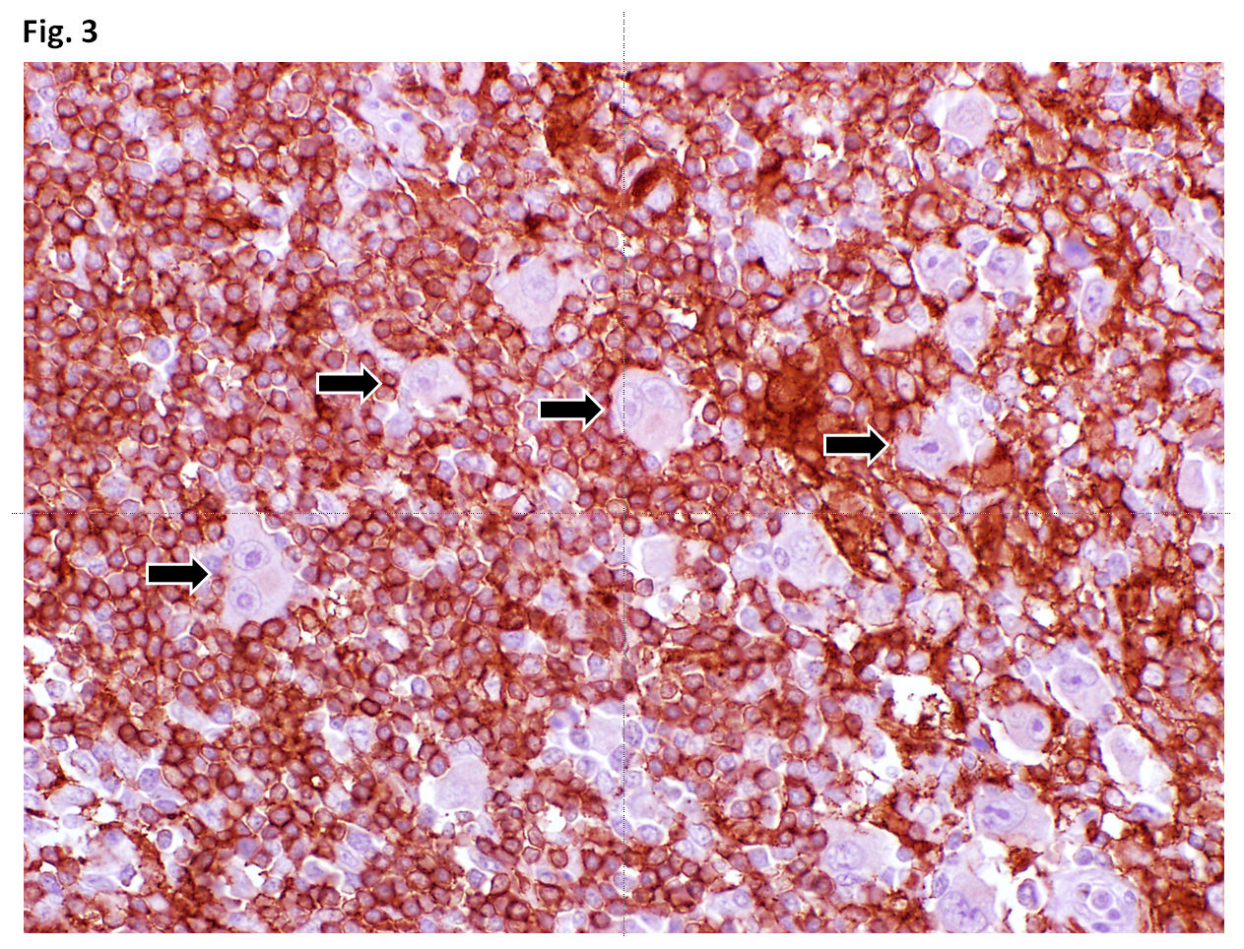
Immunohistochemical staining of the CYBB protein in cHL tissue sections. The tissue section was stained with the the primary antibody against CYBB protein. HRS cells (examples indicated by arrows) do not show staining for the protein whereas surrounding bystander cells stain strongly positive.

As *in situ* hybridisation to the *CYBB* locus in primary biopsies showed recurrent deletions of the gene in HRS cells we analysed to what extent these changes corresponded to altered CYBB protein expression. By immunohistochemistry we investigated 14 of the 18 cases studied by interphase cytogenetics for expression of the CYBB protein. Remarkably, in all of these 14 cases we observed complete loss of CYBB protein expression in all HRS cells irrespective of the presence or absence of a genomic deletion. In contrast, non-neoplastic lymphatic cells and macrophages stained positive for the CYBB protein (Figure 3). This suggests that beside deletions other mechanisms do exist in HRS cells to silence the remaining alleles and condition the observed phenotype.

### HRS cells show reduced ROS synthesis capacity

In order to functionally test the hypothesis of an impaired ROS synthesis capacity in HRS cells, we used flow cytometry to detect and quantify superoxide anion synthesis after CD30 stimulation of the cell lines analyzed. To test if CD30 stimulation induces ROS synthesis we stimulated the two CD30^+^ positive cell lines including one T-cell lymphoma and one nodular lymphocyte predominant Hodgkin lymphoma (NLPHL) cell line and observed a direct increase of superoxide anion production. As this demonstrated the usefulness of this approach, we analyzed six cHL cell lines that are characteristic for CD30 overexpression and were previously reported to have an active CD30 signalling pathway [15]. Moreover, we extended the analysis to six CD30^-^ non-Hodgkin lymphoma cell lines as negative controls (Table S1). 

**Figure 4:**
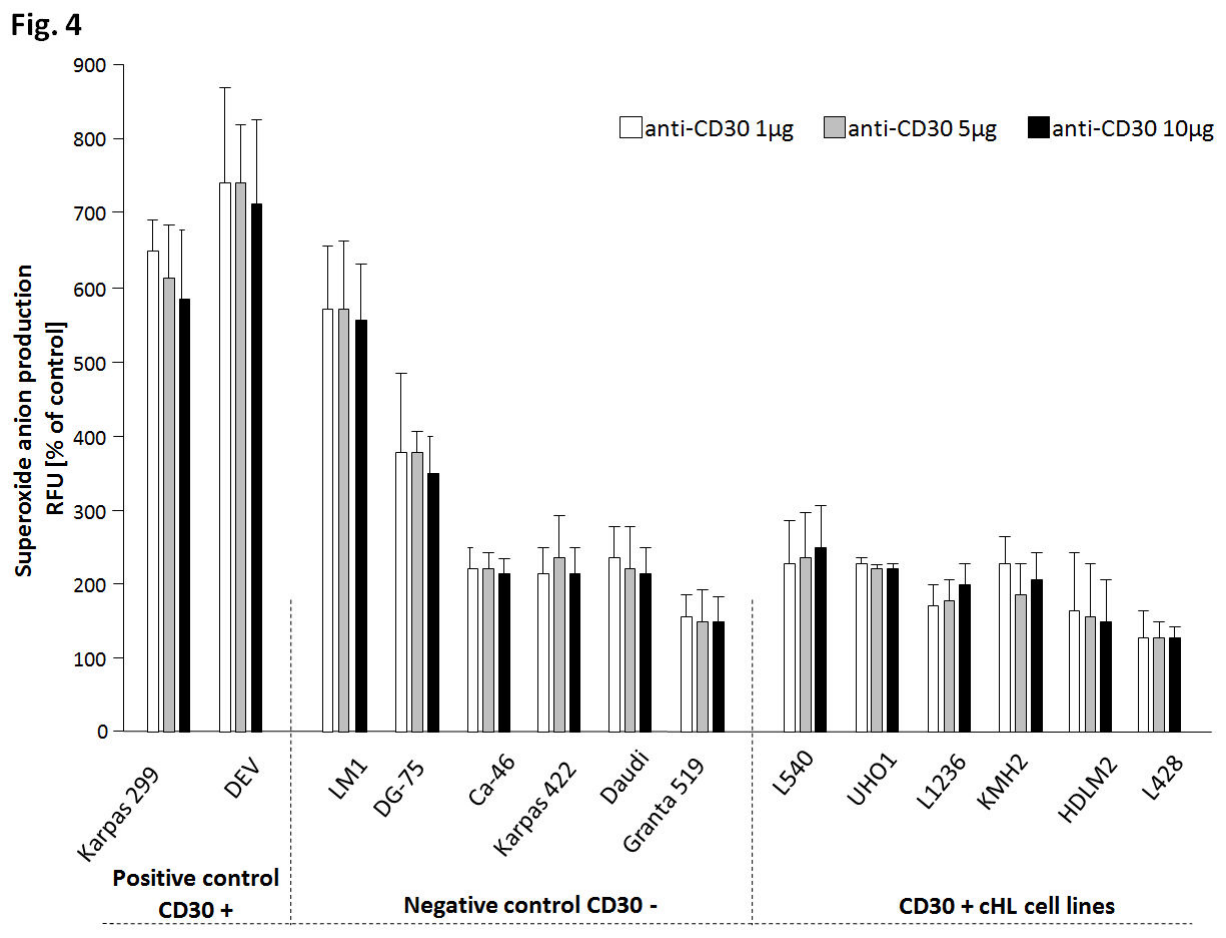
Functional analysis of NADPH oxidase. For the functional analysis of NADPH oxidase cell lines were divided into three groups according to their CD30 status. The CD30^+^ cell lines Karpas 299 and DEV (positive control cell lines), the CD30^-^ cell lines LM1, DG-75, Ca 46, Karpas 422, Daudi, Granta 519 (negative control cell lines), and in CD30^+^ cHL cell lines L540, UHO1, L1236, KMH2, HDLM2, L428 (cHL cell line cohort). For ROS synthesis all cell lines were stimulated by incubation with an anti-CD30 antibody. Intracellular level of superoxide anion (O_2_^·-^) was determined using the oxidation-sensitive fluorescent probe DHE and measured by flow cytometry (see materials and methods section for details). The bars represent the increase of superoxide anion production after stimulation. RFUs - relative fluorescent units describe the production of superoxide anion relative to the untreated cells of each culture (100% RFUs). CD30^+^ cHL cell lines and CD30^-^ negative control cell lines show limited increase of superoxide anion production (mean 1.9-fold and 2.9-fold, respectively) in contrast to both CD30^+^ positive control cell lines showing substantial increase of superoxide anion production (mean 6.7) suggesting impaired functionality of the NADPH oxidase in cHL.

In detail, we observed a mean 6.74-fold higher superoxide anion production in the CD30^+^ non-Hodgkin lymphoma cell lines as compared to unstimulated cells. In contrast, in the groups of CD30^-^ lymphoma cell lines as well as in the CD30^+^ cHL cell lines after CD30 receptor stimulation only minor increase of superoxide anion production was observed; mean 2.9-fold and 1.9-fold respectively, as compared to untreated cultures. Noteworthy, none of the cHL CD30^+^ cell lines showed elevated superoxide anion synthesis comparable to that observed in the CD30^+^ lymphoma cell lines (Figure 4). No differences in superoxide anion production were observed depending on the applied doses of CD30.

In conclusion, these results show that the functional impairment of the NADPH oxidase and the observed lower levels of ROS are features characteristic for cHL. 

## Discussion

Non-phagocytic NADPH oxidase derived ROS are involved in modulating signalling pathways and may potentially contribute to tumor pathogenesis. In support of this hypothesis we previously reported complete loss of the *CYBB* gene in the cHL cell line KMH2 suggesting that NADPH oxidase inactivation may contribute to cHL development [12]. This prompted us to analyze the other genes encoding NADPH oxidase subunits in cHL. 

We show *CYBA*, *NCF1* and *NCF4* genes to be downregulated on mRNA level in cHL cell lines as compared to normal mature B cells. Moreover, for *CYBB* and *NCF1*, we extended these findings to primary HRS cells and analyses of protein expression. Remarkably, all 14 primary cHL cases analysed for CYBB protein expression by immunohistochemistry were negative and the complete lack of the protein was characteristic for all HRS cells. Therefore, besides deletions other mechanisms must be responsible for silencing the remaining *CYBB* alleles in these cells.

In line with the findings on *NCF1*, in our recent microarray based methylation study aimed at the identification of genes hypermethylated exclusively in cHL cell lines but not in normal mature B-cell or in other B-cell lymphomas we observed hypermethylation of the *NCF1* gene in all five cHL cell lines studied namely L428, HDLM2, KMH2, L1236 and UHO1 [16]. However, no elevated methylation was observed for the other NADPH oxidase genes excluding *CYBB* that was not present on the microarray [16]. Taken together, these data provide strong indication for an epigenetic mechanism of *NCF1* silencing and shows that NADPH oxidase encoding genes are targeted by different molecular mechanisms. In contrast to the epigenetic silencing of *NCF1*, we show here that *CYBB* and *CYBA* are frequent targets of genomic losses. Importantly, germ line mutations in any of the genes manifest in chronic granulomatous disease, showing that all NADPH oxidase subunits are crucial for its proper functionality. Thus, loss of any of the genes in cHL irrespective of the triggering mechanism will result in impaired ROS synthesis capacity that we observed in the functional assay. In detail, anti-CD30 stimulation resulted in a strong 6.74-fold increase of superoxide anion production in the control CD30^+^ cell lines (positive control) and weak 2.9-fold increase in the CD30^-^ control cell lines (negative control). The cHL cell lines in turn, despite being CD30 positive, showed only a background activation of 1.9-fold suggesting an impaired functionality of the NADPH oxidase. We interpret the weak increase of superoxide anion production in the cHL cell lines and the CD30^-^ control cell lines as an unspecific reaction of the anti-CD30 antibody Ki-1-positive tumor cell culture supernatant used for stimulation.

It has been reported that CD30 signalling causes ROS production by the mitochondrial pathway, whereas inhibitors of the  NADPH oxidase complex did not affect the ROS levels measured in this study [17]. However, this interpretation is inconclusive, because ROS levels in the study by Chandel and coworkers were measured with a dye that is not responsive to superoxide anions generated by the NADPH oxidase complex [17]. This discrepancy is further evident from their observation, that in their system also TNF did not stimulate ROS-production by activation of the NADPH oxidase complex. This is in contrast to the data of Yazdanpanah et al. [18], and other reports [19-22] having clearly demonstrated that TNF (and IL-1) stimulates ROS via the NADPH oxidase complex.

Noteworthy, two of the control cell lines in our experiment, namely LM1 and DG-75, showed an increase of superoxide anion production above the background level despite being reported to be CD30^-^. We therefore measured CD30 expression of LM1, DG-75, DAUDI, and L428 cell lines using an APC-labeled monoclonal antibody and compared the fluorescence intensities to a control antibody that was matched for isotype, concentration, and fluorochrome label (data not shown). While LM1 and DG-75 cell lines indeed showed a minimally higher CD30 labelling compared to Daudi, this difference did not explain the observed increase in ROS production of LM1 and DG-75 relative to Daudi cells. ROS formation in LM1 and DG-75 is therefore likely triggered by a CD30-independent mechanism caused by unspecific binding of the antibody. Noteworthy, none of the six cHL cell lines showed a similar increase above background level.

Interestingly, the CD30^+^ DEV cell line used in this experiment is derived from a case of NLPHL [23], a rare subtype of Hodgkin lymphoma characterized by the presence of lymphocyte predominant (LP) cells. Our results show that NADPH oxidase activity differentiates between cHL and NLPHL suggesting that in case of NLPHL the enzyme remains functional. LP cells in contrast to HRS cells in the classical form do not lose their B-cell identity [14]. Therefore, it is tempting to speculate that the observed loss of NADPH oxidase activity exclusively in cHL may contribute to its loss of the B-cell phenotype. 

In line with this hypothesis it was demonstrated that ROS signalling is necessary for normal B-cell differentiation [24]. Besides, ROS were shown to regulate the activity of histone deacetylases class II (HDACs II) [25,26] and Ehlers et al. showed that inhibition of HDACs in B-cells leads to almost complete silencing of B-cell specific genes inducing a HRS cell-like phenotype [27]. Moreover, we have recently identified the B-cell related transcription factor ETS1 to be significantly downregulated in cHL [28]. Interestingly, ETS1 was shown to function in a loop with the NADPH oxidase and in mice to regulate ROS levels via the regulation of NCF1 protein expression [29,30]. Thus, the observed loss of ETS1 in cHL may result in epigenetic silencing of the *NCF1* gene reported here.

In light of the induction of ROS by CD30 signaling in several CD30^+^ cell lines and the strong and constitutive CD30 expression in primary HRS cells of cHL, one may speculate that the inactivation or downregulation of NADPH oxidase represents a strategy of the HRS cells to escape from an overwhelming and toxic ROS production, that could otherwise impair HRS cell survival.

In summary, in this study we show multiple alterations targeting the NADPH oxidase genes and impaired functionality of the enzyme in vivo. Moreover, we suggest that the loss of ROS signaling during B-cell lineage development may potentially contribute to the loss of B-cell phenotype of HRS cells.

## Materials and Methods

### Cell lines

DNA and / or cells from seven cHL cell lines, i.e. L428, HDLM2, KMH2, L1236, SUPHD1, UHO1, L540 were obtained from Deutsche Sammlung von Mikroorganismen und Zellkulturen GmbH (DSMZ) (Braunschweig, Germany) or were kindly provided by Dr. Andreas Bräuninger (University Hospital Giessen, Germany) (cells: UHO1 [31], L540 [32]). Cell line DEV (of NLPHL origin) [33] was obtained from the Department of Genetics of the University of Groningen, the Netherlands. The non-Hodgkin cell lines DG-75, Ca 46 and Daudi (Burkitt lymphomas), Karpas 422 (diffuse large B-cell lymphoma) and Granta 519 (mantle cell lymphoma) were obtained from DSMZ, whereas LM1 (diffuse large B-cell lymphoma) [34] was kindly provided by Dr. Wilhelm Woessmann (University Hospital Giessen, Germany), Karpas 299 (histiocytic high-grade lymphoma) [35] was obtained from the II Department of Medicine (University Clinic Kiel, Germany) (Table S1). Cell lines were grown in RPMI-1640 medium with Glutamax-1 (Invitrogen, Karlsruhe, Germany), supplemented with 10% or 20% (HDLM2, SUPHD1, LM1) fetal calf serum and 100 U/ml penicillin/streptomycin at 37°C in an atmosphere containing 5% CO_2_ with the exception of Granta 519 which was cultured in DMEM medium. 

### SNP 6.0 microarray analysis of cHL cell lines

DNA from cHL cell lines L428, HDLM2, KMH2, L1236, SUPHD1, UHO1 and L540 was hybridized to genome-wide human SNP array 6.0 (Affymetrix, Santa Clara, CA, USA) as described before [36]. In detail, microarrays were washed and stained with the Fluidics Station 450 (Affymetrix) and scanned with the GeneChip Scanner 3000 (Affymetrix) using the Command Console software (Affymetrix). The Birdseed v2 algorithm was used for genotyping. Copy number analysis, loss of heterozygosity (LOH) analysis and segmentation was calculated using Genotyping Console software version 3.0.2 (Affymetrix).

### Mutation screen of the CYBB gene

The cHL cell lines L428, HDLM2, L1236, SUPHD1, UHO1 and L540 were analyzed for mutations in the *CYBB* gene. Primer sequences for the mutation screening were designed using the Primer3 v. 0.4.0 software (http://frodo.wi.mit.edu/primer3/) and are available on request. DNA genomic sequences were downloaded from the UCSC Genome Browser (www.genome.ucsc.edu). PCR products encompassing each exon together with the 5’ and 3’ splicing sites were Sanger sequenced using both the forward and reverse primer by standard procedures. The fluorograms were analyzed using the Chromas Lite 2.01 software. 

### Interphase cytogenetic analysis of the CYBB gene in primary cHL biopsies

For FICTION (Fluorescence Immunophenotyping and interphase Cytogenetic as a Tool for Investigation Of Neoplasia) the Bacterial Artificial Chromosome (BAC) probe RP11-299O2 labeled in *SpectrumGreen* (Abbott/Vysis, Downers Grove, IL, USA) spanning the *CYBB* locus together with the centromeric CEPX *SpectrumOrange* (Abbott/Vysis) probe was used as described before [37,38]. Cryosections were first incubated with a monoclonal antibody against CD30 and detected with Alexa-594-conjugated secondary antibody (Molecular Probes, Leiden, The Netherlands). Always 5-20 large, CD30^+^ cells /case were evaluated independently by two observers. 

The threshold for the detection of a deletion was arbitrarily set to 30%. In detail, a deletion was scored in two cases: (i) if the signal number of the *CYBB* probe was lower than the signal number of the CEPX probe and lower than the expected number of CEPX signals in at least 30% HRS cell nuclei / case; (ii) if the signal number of the *CYBB* probe was lower than the expected number of CEPX signals in at least 30% HRS cell nuclei / case. In the first case a deletion of the X p arm harbouring the *CYBB* locus was scored and in the second a deletion of whole chromosome X.

The expected number of CEPX signals was estimated based on the ploidy of the case and the sex of the patient. Ploidy levels of the cases were estimated by taking median signal numbers for the chromosome enumeration probes CEP6 [36] CEP10 (unpublished), CEP16 [39] and CEP17 [40].

Slides were analyzed using a Zeiss fluorescence microscope (Göttingen, Germany) equipped with appropriate filter sets (AHF, Tübingen, Germany) and documented using an ISIS imaging system (MetaSystems, Altlussheim, Germany). 

### Mining of microarray gene expression profiles of cell lines, microdissected primary HRS cells and controls

Published gene expression profiles from Affymetrix U95 array of L428, HDLM2, KMH2 and L1236 cHL cell lines and normal B-cell controls (5 x centroblasts, 5 x centrocytes, 5 x naive B-cells, 5 x memory B-cells) [13] and U133 plus 2.0 array of 12 microdissected primary HRS cells samples and normal B-cell controls (5 x memory B-cells, 5 x plasma cells, 5 x naive B-cells, 5 x centrocytes, 5 x centroblasts) [14] were used for expression analysis. Data for the respective expression tags for the *CYBA*, *CYBB*, *NCF1*, *NCF2* and *NCF4* genes was extracted and visualised using the GeneCluster 2.0 software. Relative expression of the analyzed genes across the samples was compared using t-test. The gene expression dataset is available at http://ICG.cpmc.columbia.edu/faculty.htm and http://www.ncbi.nlm.nih.gov/geo (accession no. GSE 12453, 14879, 40160).

### Western blot

The cHL cell lines L428, HDLM2, KMH2, L1236, UHO1 and L540 and control cell lines LM1, Karpas 299, DEV, DG-75, Ca-46, Karpas 422, Daudi and Granta 519 were analyzed for NCF1 protein expression. Primary anti-NCF1 / p47-phox (ab795) (Abcam, Cambridge, UK) antibody detected by the secondary anti-Goat IgG (H+L) antibody conjugated with alkaline phosphatase (Jakson ImmunoResearch, USA) was used.

Chemicals were purchased from Sigma-Aldrich (Sigma-Aldrich Chemie GmbH Munich, Germany) unless otherwise stated. Cells were harvested, washed twice with PBS (phosphate-buffered sodium) and lysed for 15 min at 4°C in lysis buffer (50 mM Tris, 150 mM NaCl, 1 mM EDTA, 0.25% sodium deoxycholate, 1 mM sodium orthovanadate, pH 7.4, 1% Nonidet P40, 1% Triton X–100, 1 mM PMSF (phenylmethylsulfonyl fluoride), protease inhibitor cocktail (Roche Diagnostics Deutschland GmbH, Mannheim, Germany) and sonicated for 5 s on ice. The homogenate cells were centrifuged at 1000 x g for 10 min at 4°C. Protein concentration of cell lysates was determined by the bicinchoninic acid method using the BCA Protein Assay Reagents (Thermo Scientific / Pierce, Waltham, USA). 

Proteins were fractionated by 12.5% SDS-PAGE and transferred to nitrocellulose membrane. Membranes were blocked for 30 min at room temperature in Tris-buffered saline containing 0.1% (v/v) Tween-20, 5% milk powder and washed twice in Tris-buffered saline containing 0.1% (v/v) Tween-20. After incubation at room temperature with primary antibodies, membranes were washed with Tris-buffered saline containing 0.1% (v/v) Tween-20 and incubated with a 1:5,000 dilution of secondary anti-mouse horseradish peroxidase conjugated antibodies for 2 h at room temperature. Membranes were washed and developed using ECL detection reagent (GE Healthcare, Munich, Germany). Developed membranes were exposed to x-ray film (GE Healthcare). Antibodies against actin (C-11) SC-1615 (Santa Cruz) were used to verify equal loading of the lanes. 

Western blot quantification was done by densitometric analysis of the scanned films using Molecular Dynamics Personal Densitometer (Molecular Dynamics, Sunnyvale, USA) and the Image Quant 5.2 software (Molecular Dynamics). Relative protein quantity in relation to actin was measured and calculated for each cell line as arbitrary units (AU). For each of the analyzed proteins 6 independent Western blots were performed and a mean value of the quantifications was calculated.

### Immunohistochemistry

Immunohistochemical staining of CYBB protein was performed using a mouse monoclonal antibody as follows. FFPE tissue section (1-2 µm) were processed for antigen retrieval by boiling in citrate puffer of pH6 in a pressure cooker for 3 minutes. Incubation by the primary antibody against CYBB (NOX2/gp91phox clone ab139371, Abcam, Cambridge/UK) (1:100 dilution) was performed for 1 h at room temperature. Immunoperoxidase staining was developed using a diaminobenzidine chromogen kit (DAKO, Glostur, Denmark). Counterstaining was done with Hemalaun.

The sections were evaluated with a Olympus BX43 microscope equipped with a CCD camera DP 72 (Olympus) and documented with CellSens Entry (Olympus) software.

### Detection and quantification of superoxide anion in cell lines

The cHL cell lines L428, HDLM2, KMH2, L1236, UHO1 and L540 and control cell lines Karpas 299, DEV, LM1, DG-75, Ca-46, Karpas 422, Daudi and Granta 519 were analyzed for superoxide anion production. To stimulate superoxide anions synthesis we used anti-CD30 antibody from Ki-1-positive tumor cell culture supernatant that was kindly provided by Dr. H.P. Hansen (Department of Internal Medicine I, University Hospital Cologne, Germany). The supernatant was purified using protein G Sepharose (GE Healthcare) and diluted on the protein G matrix (GE Healthcare) with Glycin / HCl buffer pH 2.7. The antibody was stored in phosphate-buffered solution, pH 7.2. 

Prior to stimulation, cells were harvested and diluted to a concentration of 3.10E5 cells/150 µl in fresh RPMI 1640 medium or DMEM. Cells were incubated simultaneously with anti-CD30 antibody and 0.03 mM DHE (dihydroethidium) (Invitrogen / Molecular Probes, Karlsruhe, Germany) for 30 min. at 37°C in the dark. 

Intracellular level of superoxide anion (O_2_^·-^) was determined using the oxidation-sensitive fluorescent probe DHE resulting in a color shift of the dyes as described before [41,42]. The red fluorescence was detected with the FL2 filter by the FACSCalibur flow cytometer and analyzed by the BD CellQuest Pro software (BD FACSCalibur, Becton Dickinson, Heidelberg, Germany).

Each cell line was independently analyzed in two or three replications in four CD30 concentrations: untreated, 1 µg, 5 µg and 10 µg. In each replication the fluorescence intensity was measured three to four times after 30 min incubation with anti-CD30 and mean values were calculated. The mean value of the untreated cells served as standard and was regarded as 100% RFUs (relative fluorescent unit). The % RFUs of the cell lines incubated with the anti-CD30 antibody were calculated relatively to untreated cells and presented as fold change of superoxide anion production.

The cell lines analyzed were divided into three groups: 2 CD30^+^ lymphoma cell lines as positive control; 6 CD30^-^ lymphoma cell lines as negative controls, and 6 CD30^+^ cHL cell lines (Figure 4).

### Ethics statement

According to the institutional review board of the Medical Faculty of the University of Kiel (decision number D447/10 from 16.8.2010) the authors are free to use archival material sent to the Department of Pathology, Lymph Node Registry of the University Kiel, after the diagnostic process is finished. If the specimen are used anonymized, clinical data, which were available for the diagnostic process can be used too. Obtaining new clinical data about the patients need a patient informed consent. However, the latter is not the case in our study. We only used anonymized specimen and gender and age as the only clinical variables.

## Supporting Information

Table S1 **Characteristic of cell lines used in the study.**
(DOC)
