# Supplementary material for: Hodgkin-Reed-Sternberg Cells in Classical Hodgkin Lymphoma Show Alterations of Genes Encoding the NADPH Oxidase Complex and Impaired Reactive Oxygen Species Synthesis Capacity
Source: PLoS One. 2013 Dec 23;8(12):e84928. doi: 10.1371/journal.pone.0084928 (PMC3871653; doi:10.1371/journal.pone.0084928)
Supplement: Table S1 — Characteristic of cell lines used in the study. (DOC) [file pone.0084928.s001.doc]

**Table S1**

| **Cell line name** | **Type of lymphoma** | **CD30 marker** | **Literature** |
| --- | --- | --- | --- |
| **L428** | classical Hodgkin lymphoma | **+** | [1] |
| **HDLM2** | classical Hodgkin lymphoma | **+** | [1] |
| **KMH2** | classical Hodgkin lymphoma | **+** | [1] |
| **L1236** | classical Hodgkin lymphoma | **+** | [1] |
| **SUPHD1** | classical Hodgkin lymphoma | **-** | [1] |
| **UHO1** | classical Hodgkin lymphoma | **+** | [1] |
| **L540** | classical Hodgkin lymphoma | **+** | [1] |
| **KARPAS 299** | anaplastic large cell lymphoma | **+** | [1] |
| **DEV1** | nodular lymphocyte predominant Hodgkin lymphoma | **+** | [1] |
| **LM1** | diffuse large B-cell lymphoma | **-** | [2] |
| **Granta 519** | mantle cell lymphoma | **-** | [1] |
| **Daudi** | Burkitt lymphoma | **-** | [3] |
| **Karpas 422** | diffuse large B-cell lymphoma | **-** | [4] |
| **CA46** | Burkitt lymphoma | **-** | [5] |
| **DG75** | Burkitt lymphoma | **-** | [6] |

1. Drexler HG (2010) Guide to leukemia-lymphoma cell lines. 2nd edition, Braunschweig.

2. Cerchietti L, Damm-Welk C, Vater I, Klapper W, Harder L, et al. (2011) Inhibition of anaplastic lymphoma kinase (ALK) activity provides a therapeutic approach for CLTC-ALK-positive human diffuse large B cell lymphomas. PLoS One 6: e18436.

3. Wahl AF, Klussman K, Thompson JD, Chen JH, Francisco LV, et al. (2002) The anti-CD30 monoclonal antibody SGN-30 promotes growth arrest and DNA fragmentation in vitro and affects antitumor activity in models of Hodgkin's disease. Cancer Res 62: 3736-42.

4. Gruss HJ, Boiani N, Williams DE, Armitage RJ, Smith CA, et al. (1994) Pleiotropic effects of the CD30 ligand on CD30-expressing cells and lymphoma cell lines. Blood 83: 2045-56.

5. Wellmann A, Thieblemont C, Pittaluga S, Sakai A, Jaffe ES, et al. (2000) Detection of differentially expressed genes in lymphomas using cDNA arrays: identification of clusterin as a new diagnostic marker for anaplastic large-cell lymphomas. Blood 96: 398-404.

6. Dürkop H, Oberbarnscheidt M, Latza U, Bulfone-Paus S, Hirsch B, et al. (2000) The restricted expression pattern of the Hodgkin's lymphoma-associated cytokine receptor CD30 is regulated by a minimal promoter. J Pathol 192: 182-93.
